# Ventricular anti-arrhythmic effects of heptanol in hypokalaemic, Langendorff-perfused mouse hearts

**DOI:** 10.3892/br.2016.577

**Published:** 2016-01-21

**Authors:** GARY TSE, VIVIAN TSE, JIE MING YEO

**Affiliations:** 1 School of Biomedical Sciences, Li Ka Shing Faculty of Medicine, University of Hong Kong, Hong Kong, SAR, P.R. China; 2 Department of Physiology, McGill University, Montreal, Quebec H3G 1YG, Canada; 3 School of Medicine, Imperial College London, SW7 2AZ London, UK

**Keywords:** heptanol, mouse, ventricular arrhythmia, hypokalaemia, gap junction, sodium channel

## Abstract

Ventricular arrhythmic and electrophysiological properties were examined during normokalaemia (5.2 mM [K^+^]), hypokalaemia (3 mM [K^+^]) or hypokalaemia in the presence of 0.1 or 2 mM heptanol in Langendorff-perfused mouse hearts. Left ventricular epicardial or endocardial monophasic action potential recordings were obtained during right ventricular pacing. Hypokalaemia induced ventricular premature beats (VPBs) in 5 of 7 and ventricular tachycardia (VT) in 6 of 7 hearts (P<0.01), prolonged action potential durations (APD_90_) from 36.2±1.7 to 55.7±2.0 msec (P<0.01) and shortened ventricular effective refractory periods (VERPs) from 44.5±4.0 to 28.9±3.8 msec (P<0.01) without altering conduction velocities (CVs) (0.17±0.01 m/sec, P>0.05), reducing excitation wavelengths (λ, CV × VERP) from 7.9±1.1 to 5.1±0.3 mm (P<0.05) while increasing critical intervals (CI, APD_90_-VERP) from −8.3±4.3 to 26.9±2.0 msec (P>0.001). Heptanol (0.1 mM) prevented VT, restored effective refractory period (ERP) to 45.2±2.9 msec without altering CV or APD, returning λ to control values (P>0.05) and CI to 8.4±3.8 msec (P<0.05). Heptanol (2 mM) prevented VPBs and VT, increased ERP to 67.7±7.6 msec (P<0.05), and reduced CV to 0.11±0.1 m/sec (P<0.001) without altering APD (P>0.05), returning λ and CI to control values (P>0.05). Anti-arrhythmic effects of heptanol during hypokalaemia were explicable by ERP changes, scaling λ and CI.

## Introduction

Cardiac excitation involves an orderly sequence of action potential activation and recovery, and subsequently, its conduction through successive myocardial regions via gap junctions (1–3). Disruption of these processes, through alterations in conduction velocity (CV), effective refractory period (ERP) or action potential duration (APD), can result in ventricular arrhythmias. This can be exemplified by hypokalaemia, a commonly encountered condition in clinical practice and an acquired cause of long QT syndrome (4). Prolongations in the electrocardiographic QT interval reflect increases in APDs, in turn predisposing to a particular form of ventricular tachycardia known as torsades de pointes (5).

A strategy used to prevent such adverse rhythms is to increase the ERP. This can be achieved using drugs that act on various cardiac ion channels in the cell membrane. However, certain anti-arrhythmic agents can have the undesired and paradoxical effects of themselves inducing arrhythmias (6) through mechanisms such as APD prolongation (7) or CV slowing (8). Therefore, there is a requirement to develop novel drugs with improved safety profiles (9). One potential method is to target intercellular communication, which is mediated by gap junctions. For example, gap junction openers such as rotigaptide (10), and inhibitors such as carbenaloxone (11), have demonstrated promising results of preventing ventricular arrhythmias induced by ischaemia in dogs.

Mouse hearts have been extensively used to model arrhythmogenesis due to their amenability for pharmacological and genetic manipulation (12). In particular, the Langendorff perfusion system has the benefit of containing all myocardial cell types with intact intercellular coupling (13). Hypokalaemia has previously been shown to elicit frequent early after-depolarisation phenomena and sustained ventricular tachy-arrhythmias, with prolonged APDs and reduced ERPs implicated as the underlying re-entrant substrates (14,15). Heptanol is a specific gap junction inhibitor when <1 mM (16,17), but also blocks sodium channels at ≥2 mM (16,18). Previous experiments have demonstrated ventricular tachy-arrhythmias occurring at 2 mM heptanol, which was previously attributed to its effect on slowing CV despite simultaneously increasing ERP and leaving APD unaltered (19). At 0.1 mM, it had no pro-arrhythmic effects, increased ERP without altering CV or APD. The present study hypothesised that combining hypokalaemia and heptanol may paradoxically prevent ventricular arrhythmogenesis. This may be explained through heptanol reversing the ERP changes produced by hypokalaemia. However, VPBs and ventricular tachycardia (VT) as well as the electrophysiological parameters observed during hypokalaemia may be affected differently at 0.1 and 2 mM heptanol due to their differing ion channel specificities.

The present experiments first confirmed previous observations of VPBs and VT during hypokalaemic conditions. Heptanol at 0.1 mM prevented VT whilst returning ERPs to normokalaemic values without affecting APDs or CVs. By contrast, at 2 mM, it prevented VPBs and VT, associated with increased ERPs, despite reduced CVs and no further changes to APDs. The present results suggest that the net effect of a pharmacological agent on arrhythmogenicity is dependent upon the association between ERP, CV and APD, and that changes in a single parameter are sufficient to influence the arrhythmic outcome. It appears that wavelength shortening consistently predicted the occurrence of VT, implying that re-entry may be an underlying mechanism. Gap junction inhibitors, not just its openers, have protective effects against arrhythmias and their therapeutic effects warrant further exploration.

## Materials and methods

### 

#### Solutions

The experiments described in the present study used Krebs-Henseleit solution (119 mM NaCl, 25 mM NaHCO_3_, 4 mM KCl, 1.2 mM KH_2_PO_4_, 1 mM MgCl_2_, 1.8 mM CaCl_2_, 10 mM glucose and 2 mM sodium pyruvate) (pH 7.4) that had been bicarbonate-buffered and bubbled with 95% O_2_-5% CO_2_ (20). Hypokalaemic solution was prepared by decreasing the amount of KCl added to produce a final [K^+^] of 3 mM.

#### Preparation of Langendorff-perfused mouse hearts

In total, 14 mice (wild-type, 129 genetic background) between 5 and 7 months of age were used in the study. These mice were maintained in an animal facility at room temperature (21±1°C), subject to a 12:12 h light/dark cycle with free access to sterile rodent chow and water. All the experiments described here complied with the UK Animals (Scientific Procedures) Act 1986. The procedures for the preparation of Langendorff-perfused mouse hearts are as follows. Mice were sacrificed by cervical dislocation in accordance with Sections 1(c) and 2 of Schedule 1 of the UK Animals (Scientific Procedures) Act 1986. The hearts were rapidly excised and immediately submerged in ice-cold Krebs-Henseleit solution. Cannulation of the aorta was achieved using a tailor-made 21-gauge cannula that had been prefilled with ice-cold buffer. Using a micro-aneurysm clip (Harvard Apparatus, Kent, UK), the heart was securely attached to the perfusion system. Retrograde perfusion was initiated at a rate of 2–2.5 ml min^−1^ using a peristaltic pump (Watson-Marlow Bredel pumps model 505S; Falmouth, Cornwall, UK) with the perfusate passing through 200 and 5 µm filters successively and heated to 37°C using a water jacket and circulator before reaching the aorta. The hearts that regained their pink colour and spontaneous rhythmic activity were studied further (~90%). The remaining 10% were discarded. Perfusion took place for a further 20 min to minimise any residual effects of catecholamine released endogenously, prior to studying the electrophysiology of the perfused hearts.

#### Stimulation protocols

Electrical stimulation was achieved using paired platinum electrodes (1 mm interpole distance) placed at the basal right ventricular epicardium. This occurred at 8 Hz, using square wave pulses that were 2 msec in duration, with a stimulation voltage set to thrice the diastolic threshold (Grass S48 stimulator; Grass-Telefactor, Slough, UK) immediately following the start of perfusion. Programmed electrical stimulation (PES) using the S1S2 protocol was used to assess arrhythmogenicity and identify re-entrant substrates. This consisted of a drive train of eight regularly paced S1 stimuli separated by a 125 msec basic cycle length (BCL), followed by premature S2 extra-stimuli every ninth stimulus. S1S2 intervals first equalled the pacing interval and following this were successively reduced by 1 msec with each nine stimulus cycle until arrhythmic activity was initiated or refractoriness was reached, whereupon the S2 stimulus elicited no response.

#### Recording procedures

Recordings of the monophasic action potentials (MAPs) from the left ventricular epicardium were obtained using a MAP electrode (Linton Instruments, Harvard Apparatus). MAPs from the left ventricular endocardium were obtained using a custom-made MAP electrode that was made from two strands of 0.25 mm Teflon-coated silver wire (99.99% purity; Advent Research Materials, Oxfordshire, UK). The tips of the electrode were galvanically-chlorided to eliminate DC offset. The endocardial electrode was introduced through a small access window made in the inter-ventricular septum and subsequently positioned on the lateral wall of the left ventricular cavity. The positions for stimulating and recording electrodes were maintained at constant positions, with a constant inter-electrode distance of 3 mm. This allowed conduction velocities to be determined from the activation latencies. All recordings were performed using a BCL of 125 msec (8 Hz) to exclude rate-dependent differences in APDs. MAPs were pre-amplified using a NL100AK head stage, amplified with a NL104A amplifier and band pass filtered between 0.5 Hz and 1 kHz using a NL125/6 filter (Neurolog, Hertfordshire, UK) and subsequently digitized (1401plus MKII; Cambridge Electronic Design, Cambridge, UK) at 5 kHz. The samples were analysed using Spike2 software (Cambridge Electronic Design). MAP waveforms that did not match the previous established stringent criteria for MAP signals (21) were rejected. They must have stable baselines, fast upstrokes, with no inflections or negative spikes, and a rapid first phase of repolarization. Repolarization of 0% was measured at the peak of the MAP and 100% repolarization was measured at the point of return of the potential to baseline (21–23).

The following parameters were obtained from the experimental records: i) Activation latency, defined as the time difference between the stimulus and the peak of the MAP; and ii) CV, as the ratio of the inter-electrode distance to the activation latency. As the latter distance was kept constant, CVs were inversely proportional to the corresponding activation latencies; iii) ERP, defined as the longest S1S2 interval at which the S2 extrastimulus failed to initiate a ventricular signal during PES; iv) APD_x_, the time difference between the peak of the MAP and x=30, 50, 70 and 90% repolarisation; v) ΔAPD_x_, given by the endocardial - epicardial difference; vi) local critical intervals for re-excitation at the epicardium or endocardium, given by APD_90_-ERP; vii) transmural critical interval for re-excitation of the endocardium by the epicardium given by (epicardial activation latency + epicardial APD_90_) - [endocardial activation latency + endocardial ventricular effective refractory periods (VERP)], and of the epicardium by the endocardium given by (endocardial activation latency + endocardial APD_90_) - (epicardial activation latency + epicardial VERP) (15); viii) excitation wavelength given by CV × ERP; ix) APD_90_ restitution gradient obtained from restitution curves plotting APD_90_ against the previous diastolic interval (DI), assuming its maximum value at the shortest S1S2 interval studied; x) critical DI, DI_crit_, defined as the DI at which the gradient of the APD_90_ restitution curve reaches unity; xi) maximum APD_90_ reduction, a measure of APD_90_ restitution heterogeneity, defined as the maximum APD_90_ reduction observed between the longest and shortest S1S2 intervals achieved during PES; xii) CV restitution gradient obtained from restitution curves plotting CV against the previous DI, assuming its maximal value at the shortest S1S2 interval studied; xiii) CV restitution curve time constant, τ; xiv) maximum CV reduction, a measure of CV restitution heterogeneity, defined as the maximum CV reduction observed between the longest and shortest S1S2 interval achieved during PES (24).

#### Statistical analysis

All the values are expressed as mean ± standard error of the mean. Different experimental groups were compared by one-way analysis of variance (ANOVA) and Student's t-test as appropriate. P<0.05 was considered to indicate a statistically significant difference. Categorical data were compared with Fisher's exact test (one-tailed), with P<0.05 considered to indicate a statistically significant difference.

## Results

### 

#### Ventricular arrhythmogenicity, action potential activation and recovery properties

Ventricular arrhythmogenicity and its association with action potential activation and recovery properties were examined under normokalaemic conditions (5.2 mM [K^+^]_o_), hypokalaemia alone (3 mM [K^+^]_o_) and hypokalaemia in the presence of 0.1 or 2 mM heptanol in Langendorff-perfused mouse hearts. The right ventricular epicardium was electrically stimulated using either regular 8 Hz pacing or PES (25,26). MAP recordings were obtained from the left ventricular epicardium or endocardium. The epicardial stimulating and recording electrodes were separated by a constant distance of 3 mm, which permitted CVs to be calculated from the respective activation latencies and therefore enabled their comparisons between experiments. A VPB was defined as an action potential occurring prior to full repolarisation, and VT was defined as a succession of five or more action potentials at intervals closer than the BCL.

#### Concentrations of 0.1 and 2 mM heptanol exert anti-arrhythmic effects under hypokalaemic conditions

**Figure 1.**
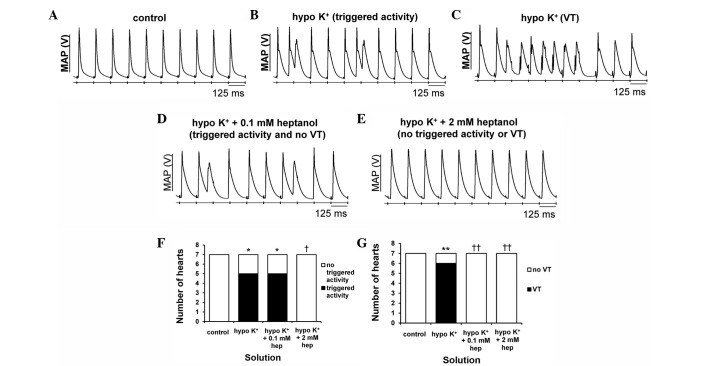
Representative traces of epicardial monophasic action potential (MAP) recordings obtained during regular 8 Hz pacing. (A) A typical regular rhythm with each MAP occurring directly after its preceding stimulus can be observed under control conditions (5.2 mM [K^+^]). (B) Hypokalaemia (3 mM [K^+^]) produced both ventricular premature beats (VPBs) and (C) ventricular tachycardia (VT). (D) Heptanol at 0.1 mM prevented VT but did not affect VPBs, (E) whereas at 2 mM prevented VPBs and VT during hypokalaemic conditions. (F) The proportion of hearts showing VPBs was significantly increased by hypokalaemia (Fisher's exact test, *P<0.05). This was unaltered by heptanol at 0.1 mM (P>0.05), but subsequently decreased at 2 mM (^†^P<0.05). (G) The proportion of hearts showing VT was significantly increased by hypokalaemia (**P<0.01) and subsequently decreased by 0.1 and 2 mM heptanol (^††^P<0.01).

The initial experiments conducted during regular pacing demonstrated consistent ventricular activity in the absence of spontaneous arrhythmias in all of the seven hearts studied under normokalaemic conditions (Fig. 1A). By contrast, VPBs (Fig. 1B) occurred in 5 hearts (Fisher's exact test, P<0.05) and VT (Fig. 1C) in 6 hearts (P<0.01) during hypokalaemia, as summarised in Fig. 1F and G, respectively. To test the effects of heptanol, it was first applied at 0.1 mM under hypokalaemic conditions and was found to prevent VT in 6 hearts (P<0.01) without altering the incidence of VPBs (P>0.05) (Fig. 1D). Raising its concentration to 2 mM prevented both VPBs in 5 hearts and VT in 6 hearts, respectively (P<0.05) (Fig. 1E).

**Figure 2.**
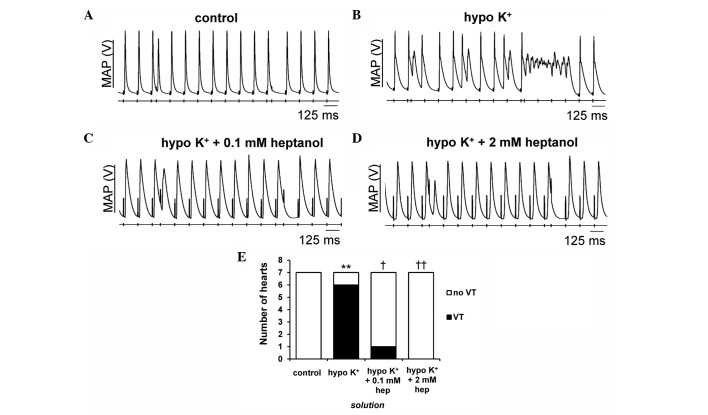
Representative monophasic action potential (MAP) recordings obtained during programmed electrical stimulation. (A) There was no evidence of provoked arrhythmias under control conditions. By contrast, (B) ventricular tachycardia (VT) was observed in under hypokalaemic conditions and inhibited by (C) 0.1 and (D) 2 mM heptanol. (E) The proportion of hearts showing provoked VT was significantly increased by hypokalaemia from 0/7 to 6/7 hearts (Fisher's exact test, **P<0.01). This was reduced to 1/7 and 0/7 hearts following further introduction of 0.1 (^†^P<0.05) and 2 mM (^††^P<0.01) heptanol, respectively.

The second set of experiments explored for re-entrant substrates using PES, imposing extrasystolic S2 stimuli following trains of regular S1 pacing stimuli. The S1S2 interval was initially at the BCL and subsequently reduced by 1 msec with each cycle until the S2 stimuli produced either arrhythmic activity or refractoriness, with the latter indicating that the VERP had been reached. None of the hearts studied demonstrated evidence of arrhythmias under normokalaemic conditions (Fig. 2A; incidence summarised in Fig. 2E). By contrast, VT was induced by PES in 6/7 hearts during hypokalaemia (Fig. 2B) (P<0.01). Introduction of 0.1 (Fig. 2C) and 2 mM (Fig. 2D) heptanol prevented VT from occurring in 5 (P<0.05) and 6 out of these 7 hearts (P<0.01), respectively.

#### Anti-arrhythmic effects of heptanol can be explained by VERP changes despite abnormal CVs and APDs

Previous studies using animal models of hypokalaemia have associated its pro-arrhythmic effects with reduced CVs (27), prolonged epicardial APDs, and prolonged or unaltered endocardial APDs, leading to decreased ΔAPDs given by the epicardial-endocardial difference (14) and decreased VERPs (15). By contrast, pro-arrhythmic effects of heptanol have been attributed to reduced CVs despite increased VERPs and normal APDs (19). To quantify the effect of heptanol on hypokalaemia-induced arrhythmogenesis, these parameters were determined from the data obtained in the aforementioned experiments (n=7) and correlated with the arrhythmogenicity findings.

**Figure 3.**
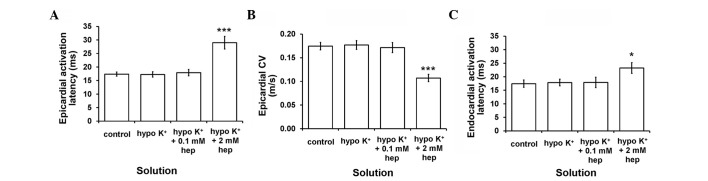
(A) Epicardial activation latency and (B) conduction velocity (CV), and (C) endocardial activation latency obtained during regular 8 Hz pacing. These were not altered by hypokalaemia whether prior or subsequent to introduction of 0.1 mM heptanol (analysis of variance, P>0.05). By contrast, epicardial (***P<0.001) and endocardial (*P<0.05) activation latencies were significantly increased by 2 mM heptanol, corresponding to reduced CVs (***P<0.001). The epicardial activation latencies were not significantly different from the corresponding endocardial activation latencies under all pharmacological conditions studied (P>0.05).

Firstly, epicardial activation latency was 17.4±0.8 msec (Fig. 3A) under normokalaemic conditions, corresponding to a CV of 0.17±0.01 m/sec (Fig. 3B), while endocardial activation latency was 17.4±1.4 msec (Fig. 3C). These values were not altered by hypokalaemia alone or 0.1 mM heptanol (ANOVA, P>0.05). By contrast, 2 mM heptanol increased epicardial and endocardial activation latencies to 29.0±2.3 msec (P<0.001) and 23.2±2.0 msec (P<0.05), respectively, and therefore decreased CVs to 0.11±0.01 m/sec (P<0.001). Epicardial activation latencies were not significantly different from their corresponding endocardial activation latencies under any of the aforementioned pharmacological conditions studied (P>0.05).

**Figure 4.**
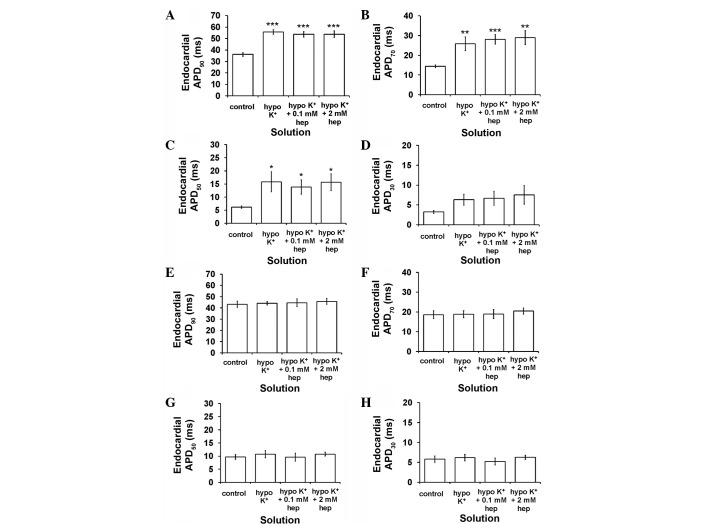
Epicardial action potential durations (APD_x_) at (A) x=90, (B) 70, (C) 50 and (D) 30% repolarisation obtained during regular 8 Hz pacing. Hypokalaemia increased epicardial APD_90_ (ANOVA, ***P<0.001), APD_70_ (**P<0.01) and APD_50_ (*P<0.05) values but left APD_30_ unaltered (P>0.05). 0.1 and 2 mM heptanol did not further alter these values (P>0.05). Endocardial APD_x_ at (E) x=90, (F) 70, (G) 50 and (H) 30% repolarisation obtained during regular 8 Hz pacing. Hypokalaemia treatment whether prior or subsequent to introduction of 0.1 mM and 2 mM heptanol did not further alter epicardial APD_90_, APD_70_, APD_50_ and APD_30_ (ANOVA, P>0.05). ANOVA, analysis of variance.

**Figure 5.**
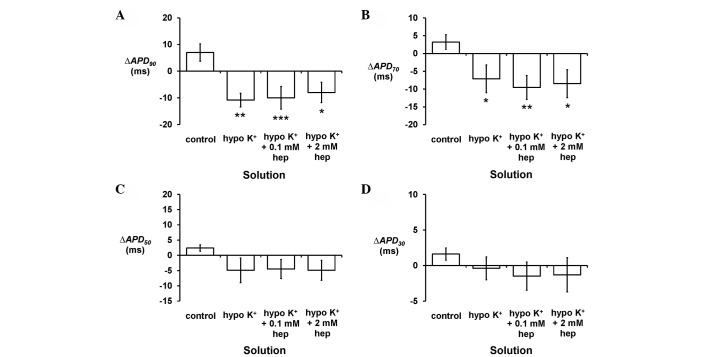
ΔAPD_x_ given by endocardial APD_x_ - epicardial APD_x_ at (A) x=90, (B) 70, (C) 50 and (D) 30% repolarisation obtained during regular 8 Hz pacing. Hypokalaemia decreased ΔAPD_90_ (Student's t-test), ΔAPD_70_, but ΔAPD_50_ and ΔAPD_30_ were unaltered. These values were not further altered by 0.1 or 2 mM heptanol (P>0.05). APD, action potential duration. (*P<0.05, **P<0.01, ***P<0.001).

Secondly, epicardial APD_90_ was increased from 36.2±1.7 to 55.7±2.0 msec by hypokalaemia (P<0.001) (Fig. 4A), as were APD_70_ (P<0.01) (Fig. 4B) and APD_50_ (P<0.05) (Fig. 4C) but not APD_30_ (P>0.05) (Fig. 4D). By contrast, endocardial APD_90_ (Fig. 4E), APD70 (Fig. 4F), APD_50_ (Fig. 4G) and APD_30_ (Fig. 4H) (P>0.05) were unaltered. These findings corresponded to decreased ΔAPD_90_ (P<0.01) (Fig. 5A) and ΔAPD_70_ (P<0.05) (Fig. 5B), but unaltered ΔAPD_50_ and ΔAPD_30_ (P>0.05) (Fig. 5C and D, respectively). Neither 0.1 nor 2 mM heptanol further altered any of the APD_x_ and therefore ΔAPD_x_ (P>0.05 in all cases).

**Figure 6.**
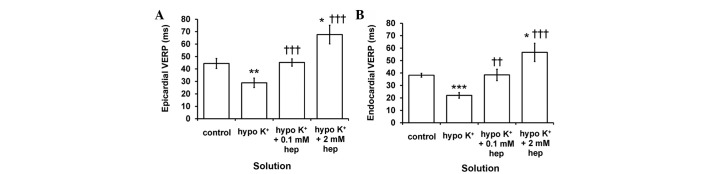
(A) Epicardial and (B) endocardial ventricular effective refractory periods (VERPs) obtained during programmed electrical stimulation. Significant differences from *normokalaemic and ^†^hypokalaemic values, respectively. Epicardial and endocardial VERPs were significantly reduced by hypokalaemia (analysis of variance, **P<0.01 and ***P<0.001 for epicardium and endocardium, respectively), but restored to normokalaemic values by 0.1 mM heptanol (P>0.05). These were further increased by 2 mM heptanol (*P<0.05). (^††^P<0.01, ^†††^P<0.001).

Finally, epicardial (Fig. 6A) and endocardial (Fig. 6B) VERPs were decreased from 44.5±4.0 to 28.9±3.8 msec (P<0.01) and from 38.2±1.6 to 22.0±2.3 msec (P<0.001), respectively, during hypokalaemia. However, these were restored to 45.2±2.9 and 38.5±4.6 msec, respectively (P<0.001 and P<0.01), by 0.1 mM heptanol, which were indistinguishable from values obtained under normokalaemic conditions. By contrast, they were increased to 67.7±7.6 (P<0.05) and 56.6±7.4 msec (P<0.05), respectively, at a higher concentration of 2 mM. Epicardial VERPs were significantly greater than the corresponding endocardial VERPs in the presence of 2 mM heptanol (P<0.05), but not under any other pharmacological conditions (P>0.05).

#### Anti-arrhythmic effects of heptanol correlate directly with critical intervals and excitation wavelengths

Increases in critical intervals (APD_90_-VERP) either locally or across the myocardial wall (15) and decreases in excitation wavelengths (CV × VERP) (19,28) have been associated with increased arrhythmic tendency. The local critical intervals for the epicardium and endocardium are given by epicardial APD_90_ - epicardial VERP and endocardial APD_90_ - endocardial VERP, respectively. The critical interval for transmural re-excitation of the epicardium by the endocardium is given by (endocardial latency + endocardial APD_90_) - (epicardial latency + epicardial VERP), and that of the endocardium by the epicardium is given by (epicardial latency + epicardial APD_90_) - (endocardial latency + endocardial VERP).

**Figure 7.**
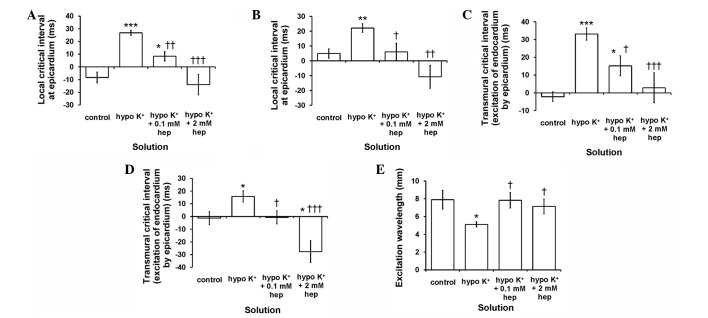
(A-D) Critical intervals [action potential durations (APD)_90_ - ventricular effective refractory periods (VERP)] and (E) excitation wavelength [conduction velocity (CV) × ventricular effective refractory periods (VERP)]. Significant differences from *normokalaemic and ^†^hypokalaemic values, respectively. Local critical intervals obtained from the (A) epicardium were significantly increased by hypokalaemia (Student's t-test, ***P<0.001) and subsequently reduced by 0.1 mM (^††^P<0.01; ^†^P<0.05) and 2 mM heptanol (^†††^P<0.001). Those obtained from the (B) endocardium, and transmural critical intervals for re-excitation of the endocardium by the (C) epicardium and that of the epicardium by the (D) endocardium exhibited similar patterns of changes. (E) Excitation wavelength was significantly reduced by hypokalaemia (analysis of variance, *P<0.05) but returned to normokalaemic values following introduction of 0.1 or 2 mM heptanol (P>0.05).

The local critical intervals obtained from the epicardium (Fig. 7A) were found to increase from −8.3±4.3 to 26.9±2.0 msec by hypokalaemia (P<0.001). These were subsequently reduced by 0.1 mM (P<0.05) and restored to normokalaemic values by 2 mM heptanol (P>0.05). Similar patterns of changes were observed for local critical intervals obtained from the endocardium (Fig. 7B), and for transmural critical intervals reflecting the period permitting re-excitation of the endocardium by the epicardium (Fig. 7C) and of the epicardium by the endocardium (Fig. 7D). Excitation wavelengths (Fig. 7E) were significantly reduced from 7.9±1.1 to 5.1±0.3 mm by hypokalaemia (P<0.05), and returned to normokalaemic values by 0.1 and 2 mM heptanol (P>0.05).

#### Heptanol does not alter APD or CV restitution properties during hypokalaemia

Previous studies have associated increased arrhythmogenicity with increases in maximum APD_90_ restitution gradients, critical diastolic intervals (DIs and DI_crit_), and maximum APD_90_ reduction between the longest and shortest S1S2 intervals studied under hypokalaemic conditions (29). In other models systems, by contrast, increased arrhythmogenicity was attributed to abnormal CV restitution. Thus, increased maximum CV restitution gradients were observed in untreated rabbit hearts (30); increased time constants of the fitted restitution curves (i.e., decreased CV restitution gradients) was demonstrated in ion channel models and diacetyl monoxime-treated rabbit hearts (31,32); increase in maximal CV reductions between the longest and shortest S1S2 intervals studied was seen in D600-treated rabbit hearts (24).

In the present study, restitution curves were constructed using the PES data obtained above, by plotting APD_90_ or CV against the preceding DI, and were then fitted with an exponential function of the form y=y_0_+Ae^−x/τ^ by a least-squares method using a Levenberg-Marquardt algorithm. y represents either APD_90_ or CV, and × represents DI, whereas y_0_, A and τ are constants. The gradient is given by

$$\frac{\text{dy}}{\text{dx}}=\frac{A}{\tau }e^{-x/\tau }$$

assuming its maximal value at the shortest S1S2 interval reached during PES. DI_crit_ was defined as the DI at which the gradient of the fitted function reached unity. Maximum APD_90_ or CV reduction, reflecting heterogeneity in restitution, was defined as the difference between values obtained at the longest S1S2 interval and those obtained at the shortest S1S2 interval.

**Figure 8.**
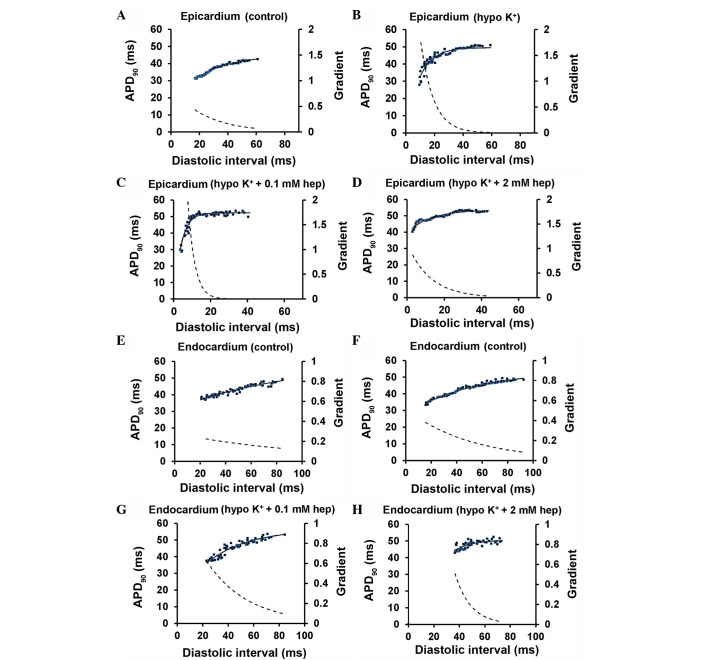
Restitution curves plotting action potential durations (APD)_90_ against preceding diastolic interval (DI) obtained from the (A-D) epicardium and (E-H) endocardium under control and hypokalaemic conditions prior and subsequent to the introduction of 0.1 or 2 mM heptanol. Curves were fitted with mono-exponential growth functions obtained by least-squares fitting to the values of APD_90_ and DI (solid lines, left ordinates). Gradients were obtained by differentiation of the fitted functions (broken lines, right axes).

Fig. 8 shows examples of APD_90_ restitution curves (solid lines, left ordinates) and their gradients (broken lines, right axes) obtained at the epicardium (Fig. 8A–D) and the endocardium (Fig. 8E–H) under normokalaemic and hypokalaemic conditions prior and subsequent to the introduction of 0.1 or 2 mM heptanol; fitted parameters summarised in Tables I and II, respectively. APD_90_ decreased with decreasing DIs under all pharmacological conditions studied.

**Figure 9.**
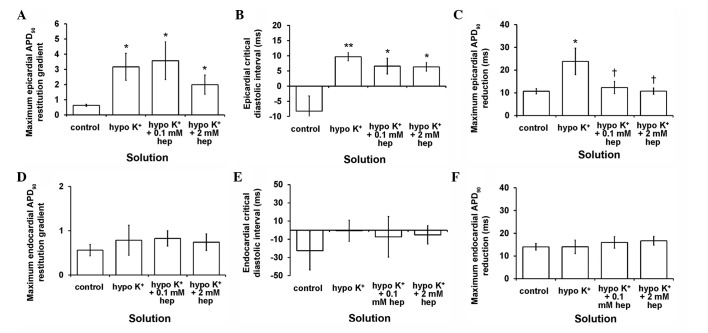
Maximum action potential durations (APD)_90_ restitution gradients, critical diastolic intervals and maximum APD_90_ reductions obtained from the (A-C) epicardium and (D-F) endocardium. All three parameters were increased by hypokalaemia (*P<0.05, **P<0.01 and *P<0.05, respectively). Of these, maximum APD_90_ reductions were restored to normokalaemic values (P>0.05), whereas maximum APD_90_ restitution gradients and critical diastolic intervals were not further altered by 0.1 or 2 mM heptanol. All three parameters obtained from the endocardium were unaltered by hypokalaemia whether prior and subsequent to the introduction of 0.1 or 2 mM heptanol (P>0.05 in all cases). (^†^P<0.05).

The maximal APD_90_ restitution gradients (Fig. 9A), DI_crit_ (Fig. 9B) and maximum APD_90_ reductions (Fig. 9C) in the epicardium were increased by hypokalaemia (P<0.05, P<0.01 and P<0.05, respectively). Introduction of 0.1 or 2 mM heptanol restored maximum APD_90_ reductions to normokalaemic values (P>0.05), but did not further alter maximum APD_90_ restitution gradients or DI_crit_. All these corresponding parameters in the endocardium remained unaltered (Fig. 9D–F) during hypokalaemia conditions whether prior or subsequent to introduction of 0.1 or 2 mM heptanol (P>0.05 in all cases).

**Figure 10.**
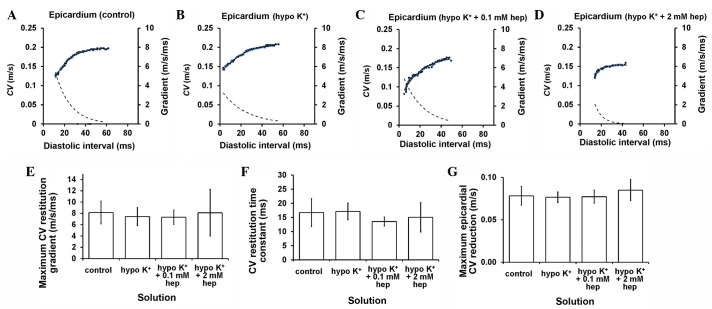
Restitution curves plotting conduction velocity (CV) against preceding diastolic interval (DI) obtained under (A) control and hypokalaemic conditions (B) prior and subsequent to the introduction of (C) 0.1 or (D) 2 mM heptanol. Curves were fitted with mono-exponential growth functions obtained by least-squares fitting to the values of CV and DI (solid lines, left ordinates). Gradients were obtained by differentiation of the fitted functions (broken lines, right axes). (E) Maximum CV restitution gradients, (F) time constants of restitution curves and (G) maximum CV reductions. None of these parameters was altered by hypokalaemia whether prior or subsequent to the introduction of 0.1 or 2 mM heptanol (P>0.05).

Fig. 10 progresses to show examples of epicardial CV restitution curves (solid lines, left ordinates) and their gradients (broken lines, right axes) obtained under normokalaemic and hypokalaemic conditions prior and subsequent to introduction of 0.1 and 2 mM heptanol (Fig. 10A–D), with fitted parameters summarised in Table III. However, there was no difference in maximum CV restitution gradients (Fig. 10E), time constants τ of the restitution curves (Fig. 10F) or maximum CV reductions (Fig. 10G) between these pharmacological conditions (P>0.05).

Taken together, all the above findings demonstrate that hypokalaemia elicited VPBs and VT, associated with prolonged epicardial APD_90_, unaltered endocardial APD_90_, and reduced epicardial and endocardial VERPs despite unaltered activation latencies and CVs. These changes corresponded to decreased ΔAPDs, increased critical intervals and reduced excitation wavelengths. Dynamic substrates in the form of abnormal APD restitution also appeared to have a role in its arrhythmogenesis, as exemplified by increases in maximum APD_90_ restitution gradients, DI_crit_ and maximum APD_90_ reductions observed during PES. However, CV restitution did not have an apparent contribution, as maximum CV restitution gradients, time constants of CV restitution curves and maximum CV reductions all remained unchanged. Heptanol at 0.1 mM prevented VT by normalising VERPs, resulting in normal critical intervals and normal excitation wavelengths. At 2 mM, it prevented VPBs and VT by increasing VERPs despite reducing CVs, which corresponded to reduced critical intervals and increased excitation wavelengths. These anti-arrhythmic effects observed at heptanol concentrations were associated with restoration of maximum APD_90_ reductions to normokalaemic values despite no further changes to APDs or the remaining APD_90_ and CV restitution parameters.

## Discussion

Ventricular arrhythmias represent a significant cause of sudden cardiac death, accounting for around 60,000 fatalities in the UK (33), 200,000 fatalities in the US (34) and 4–5 million fatalities worldwide (35) per year. Hypokalaemia is a cause of electrocardiographic QT prolongation, reflecting delayed action potential repolarisation (4), which predisposes to a particular form of polymorphic VT termed torsade de pointes (5). It is the most common electrolyte abnormality identified in hospitalised patients (36) and therefore represents an important cause of arrhythmias observed in clinical practice (37).

Ventricular arrhythmic properties of hypokalaemic Langendorff-perfused mouse hearts were examined in the presence and absence of 0.1 or 2 mM heptanol. This is an agent that reversibly inhibits gap junctions at concentrations <1 mM and also sodium channels ≥2 mM (16,18). Previous studies have described pro-arrhythmic effects of hypokalaemia (14,15,29) or heptanol (19). Heptanol (2 mM) is known to induce ventricular tachy-arrhythmias through decreases in CVs, despite also increasing VERPs and leaving APDs unchanged under normokalaemic conditions. The central hypothesis here is that changes in VERPs produced by heptanol could compensate for prolonged APDs and thereby abolish arrhythmogenesis produced by hypokalaemia. This may be explicable by changes in critical intervals given by APD - VERP and excitation wavelengths given by CV × VERP.

Monophasic action potential (MAP) recordings were obtained from the left ventricular epicardium or endocardium. Previous studies have shown that APDs derived from such recordings reflect the time courses of the cellular action potential obtained from single cells (21,38,39). Two different stimulation protocols were used. Firstly, regular pacing at 8 Hz, close to the *in vivo* heart rate (40), was used to detect spontaneous arrhythmogenesis. This revealed VPBs and VT during hypokalaemia in an absence of alterations in CV, in agreement with previous findings (14). Heptanol (0.1 mM) prevented VT without affecting VPBs, but at 2 mM prevented both despite a reduction in CV.

Secondly, PES procedures, which delivered increasing premature S2 stimuli following trains of regularly-timed S1 stimuli, were used to detect the presence of re-entrant substrates, as has been performed in clinical practice (25). This protocol also allowed exploration of the role of CV and APD restitution in hypokalaemia-induced arrhythmogenicity (29,30,41). During hypokalaemia, an increase in the proportion of hearts showing provoked VT was observed. This was associated with prolonged epicardial, but unaltered endocardial APDs and reduced epicardial and endocardial VERPs, in an absence of alterations in CVs. Epicardial APD restitution gradients and critical diastolic intervals were increased. The difference between epicardial APD obtained from the longest S1S2 interval and APD obtained from the shortest S1S2 interval studied was increased, reflecting an increased heterogeneity in APD restitution (24). The latter finding could be explained by reduced VERPs, which would allow shorter S1S2 intervals and therefore shorter APDs to be attained. Endocardial APD restitution gradients, critical diastolic intervals and APD differences were all unaltered. CV restitution gradients, restitution time constants and restitution heterogeneity were not affected. Notably, these results obtained during extrasystolic stimulation are in agreement with previous findings that were determined by a dynamic pacing protocol (29). This confirms the value of using PES in restitution analysis, as previously discussed (42). Such a protocol has the advantage of safety over dynamic pacing in a clinical setting, which can induce myocardial ischaemia from tachycardia pacing (42).

Together, these changes resulted in a negative ∆APD_90_ given by endocardial APD_90_ - epicardial APD_90_, suggesting a reversal of transmural repolarisation gradients, which has previously been associated with VT (14). The excitation wavelength given by CV × VERP was decreased, thereby predisposing the hearts to circus-type re-entry (43). Furthermore, there was an increase in the critical interval given by APD - VERP, which would increase the likelihood of re-excitation occurring prior to full action potential repolarisation (15). This may reflect an underlying re-entrant mechanism previously termed prolonged repolarisation-dependent re-excitation (44) and phase 2 re-entry (45) described in Brugada syndrome.

Heptanol (0.1 mM) prevented the induction of VT and reversed ERP changes, despite leaving APD abnormalities and CV unaffected. This in turn led to the return of the excitation wavelength and critical interval to normokalaemic values, consistent with its effects in preventing VT. The fact that VPBs persisted can be explained by prolonged APDs despite normal VERPs. This would still permit the re-activation of L-type calcium currents, previously implicated in the development of early after-depolarisations and subsequently induced triggered activity (46–48). Heptanol (2 mM) also prevented VT, which is associated with increased ERPs. This is despite persistently prolonged APDs and an additional slowing in CV, expected to increase arrhythmogenicity. These changes corresponded to increased excitation wavelengths and decreased critical intervals, both of which would decrease arrhythmogenicity. At this concentration, heptanol also prevented VPBs, which can be explained by increased VERPs; this would prevent EADs from eliciting premature action potentials in the myocardium that has not recovered from refractoriness. In the presence of either 0.1 or 2 mM heptanol, APD restitution properties remained abnormal and CV restitution was not affected during hypokalaemia.

The above findings obtained during PES contrast with previous observations during normokalaemia in which no pro-arrhythmic effects were observed at 0.1 mM, but inducible VT was detected at 2 mM heptanol (19). These differences can be explained by 2 mM heptanol reducing CV more than it increases ERP with a consequent decrease in excitation wavelength. The anti-arrhythmic action of heptanol in hypokalaemia observed here therefore complements previous demonstrations of its other beneficial effects in reducing infarct size in mouse and rat hearts (49,50) and preventing ventricular arrhythmias induced by ischaemia in rat hearts (50,51). A limitation of the present study is that the mechanisms of VPBs and VT cannot be determined. However, hypokalaemia is known to elicit EADs and the VPBs observed may reflect underlying triggered activity. VT could be due to re-entry, although it could theoretically be a run of VPBs from focal activity. If VT is caused by re-entry, the mechanism is uncertain, whether it is due to circus-type, phase 2 or spiral wave re-entry. However, premature activation by phase 2 re-entry (or indeed a VPB from triggered activity) can in turn facilitate the induction of circus-type movement (52).

Protective effects of gap junction in reducing infarct size as well as preventing arrhythmogenesis produced by ischaemia have previously been described (49,50). Theoretical work has shown that in non-uniform tissue, mild loss of gap junction function paradoxically increases CV, thereby improving the safety margin of conduction (53). Thus, this may remove unidirectional blocks, converting them into bilateral conduction, which would protect against arrhythmogenesis (54–56). In the present study, the experiments show that in addition to the effects on conduction, the gap junction uncoupler heptanol also influences VERP and this may underlie its anti-arrhythmic action in hypokalaemia. This occurred despite a lack of correction of the repolarisation abnormalities (prolonged APD and increased APD restitution steepness), even in the presence of reduced CV, which alone is pro-arrhythmic (19).

The proof of concept demonstrated here is that although the arrhythmic risk of hypokalaemia has traditionally been associated with prolonged QT interval (57) and increased QT dispersion (58), increasing VERPs can prevent arrhythmias even when such repolarisation abnormalities persist. Heptanol (2 mM) is known to induce VT under normokalaemic conditions, at a lower concentration of 0.1 mM, however, it had no pro-arrhythmic effects (19). Its possible therapeutic effects therefore warrant further investigation.

## Figures and Tables

**Table I.** Parameters for epicardial APD_90_ restitution curves obtained during programmed electrical stimulation.

Condition	y_0_, msec	A, msec	τ, msec
Control	45.2±2.9	−23.2±3.6	21.7±4.4
Hypo K_+_	51.1±2.0	−108.3±62.6	12.6±4.5
Hypo K^+^ + 0.1 mM heptanol	55.5±2.2	−82.1±40.0	4.7±1.0
Hypo K^+^ + 2 mM heptanol	60.0±5.7	−180.1±118.0	11.2±4.8

APD, action potential duration.

**Table II.** Parameters for endocardial APD_90_ restitution curves obtained during programmed electrical stimulation.

Condition	y_0_, msec	A, msec	τ, msec
Control	43.6±3.6	−124.6±97.8	39.0±13.6
Hypo K^+^	54.3±19.8	−36.6±18.9	20.4±26.5
Hypo K^+^ + 0.1 mM heptanol	52.0±9.8	−48.5±25.0	50.9±42.4
Hypo K^+^ + 2 mM heptanol	62.5±22.3	−31.0±20.1	15.4±34.9

APD, action potential duration.

**Table III.** Parameters for conduction velocity restitution curves obtained during programmed electrical stimulation.

Condition	y_0_, m/sec	A, m/sec	τ, msec
Control	0.20±0.01	−0.62±0.420	16.7±4.9
Hypo K^+^	0.20±0.01	−0.15±0.012	17.1±3.0
Hypo K^+^ + 0.1 mM heptanol	0.16±0.01	−0.15±0.027	13.5±1.6
Hypo K^+^ + 2 mM heptanol	0.16±0.06	−0.18±0.082	15.1±5.2
